# Beta-Thalassemia Major and Myocardial Iron Overload: A Longitudinal Study with Magnetic Resonance Imaging

**DOI:** 10.1155/2024/8842016

**Published:** 2024-07-24

**Authors:** Kiara Rezaei-Kalantari, Elahe Meftah, Saeed Tofighi, Kamand Khalaj, Arezou Zoroufian, Marzieh Motevalli, Mohammed Inusah Bihinaa, Negar Omidi, Seyyed Mojtaba Ghorashi

**Affiliations:** ^1^ Rajaie Cardiovascular Medical and Research Center Iran University of Medical Sciences, Tehran, Iran; ^2^ Students' Scientific Research Center Tehran University of Medical Sciences, Tehran, Iran; ^3^ Tehran Heart Center Tehran University of Medical Sciences, Tehran, Iran; ^4^ School of Medicine Tehran University of Medical Sciences, Tehran, Iran; ^5^ Cardiac Primary Prevention Research Center Cardiovascular Diseases Research Institute Tehran University of Medical Sciences, Tehran, Iran; ^6^ Cardiovascular Disease Research Institute Tehran Heart Center Tehran University of Medical Science, Tehran, Iran

## Abstract

**Background:**

Patients with *β*-thalassemia major depend on lifelong transfusion, resulting in tissue iron overload. This longitudinal retrospective observational study aims to assess myocardial and liver iron overload using magnetic resonance imaging (MRI) and investigate the lag between myocardial and liver iron unloading in *β*-thalassemia patients undergoing chelation therapy.

**Methods:**

Beta-thalassemia major patients with at least two MRI studies between 2016 and 2020 were enrolled. Myocardial and liver iron overload were defined as T2^*∗*^ less than 20 and 2.1, respectively. Outcomes included mortality, myocardial and liver T2^*∗*^ changes, and systolic dysfunction assessed by cardiac MRI.

**Results:**

Fifty-five patients with a mean age of 24.62 ± 7.94 years, a mean follow-up duration of 24.3 ± 12.9 months, and a mean ferritin level of 1475.75 ± 771.12 ng/mL were enrolled. All of the abovementioned patients only took deferoxamine as the iron-chelating medication. Mortality occurred in three patients (5.5%) during follow-up. Liver T2^*∗*^ significantly increased (*p* value <0.05), while myocardial T2^*∗*^ showed a nonsignificant increase. Iron unloading of the myocardium was not significantly different from that of the liver and did not result in a significant lag (56% vs. 44%; *p* value = 0.419). Baseline myocardial T2^*∗*^ correlated with extramedullary hematopoiesis, weekly number of deferoxamine injections (*p* value <0.01), timing between the transfusions, and serum ferritin (*p* value <0.05).

**Conclusion:**

Liver T2^*∗*^ reduced during deferoxamine chelation therapy, while myocardial T2^*∗*^ remained unchanged. No significant lag was observed between myocardial and liver iron unloading. Further studies are required to elucidate these findings.

## 1. Introduction

Thalassemia comprises a group of disorders with impaired hemoglobin synthesis, leading to anemia and ineffective erythropoiesis. The accumulation of the unbalanced globin chain is the primary pathophysiology of anemia in thalassemia disorders and *β*-thalassemia major in particular [1]. Ineffective erythropoiesis may give rise to extramedullary hematopoiesis and symptoms related to the invasion of their adjacent anatomic location [2]. Patients with *β*-thalassemia major require lifelong transfusions [1], resulting in iron overload, complications, and mortality [3]. The primary cause of morbidity in thalassemia major is iron overload, which is a common chronic transfusion-induced side effect [4]. In this respect, the effective iron chelates have considerably reduced the mortality and morbidity rates of these patients.

Various cardiovascular complications are expected in *β*-thalassemia major, including different forms of impaired cardiac function and structure. Cardiac remodeling may occur secondary to blood volume overload, mainly as compensation for severe anemia. Cardiac siderosis may result in electrocardiogram changes and arrhythmia, particularly atrial fibrillation. Other cardiovascular complications include tricuspid regurgitation and pulmonary hypertension [5, 6].

Heart failure is one of the significant morbid cardiovascular complications in *β*-thalassemia major. Various mechanisms in *β*-thalassemia major patients induce heart failure, including hypoxia, volume overload, and microvasculopathy [7, 8]. Cardiac siderosis is the most common and critical etiology of heart failure and cardiomyopathy [6, 9]. The degree of tissue iron loading is a vital contributor to transfusion-induced iron toxicity. Tissue biopsy is the gold standard for assessing tissue iron load. However, noninvasive methods, particularly magnetic resonance imaging (MRI), provide valuable insights into tissue iron overload without invasive procedures and exposure to ionizing contrast [10, 11]. Multislice multiecho T2^*∗*^ cardiac magnetic resonance (CMR) can be employed to detect myocardial iron overload. The T2^*∗*^ threshold for iron overload myocardial dysfunction is 20 milliseconds (ms). Therefore, no myocardial dysfunction or mortality is anticipated in cardiac T2^*∗*^ over 20 ms [12, 13]. Longitudinal MRI studies would allow clinicians to quantitatively assess both myocardial and liver iron unloading, their association, and the differences in their response to iron chelation therapy. These studies can also demonstrate the state of extramedullary hematopoiesis and its association with tissue iron loading and chelation therapy. The reduced myocardial iron overload demonstrated by cardiac MRI is associated with a dramatic cardiac mortality reduction [14]. Thus, it is justifiable to perform follow-up and treatment modifications with CMR for the patients suffering from *β*-thalassemia major.

This study aims to longitudinally observe *β*-thalassemia major patients regarding iron deposition and removal in the myocardium and liver and outcomes during follow-up. The study also aims to investigate the difference in iron removal from the liver and myocardium in response to chelation therapy.

## 2. Materials and Methods

### 2.1. Study Design

This longitudinal retrospective observational study was conducted from April 2016 to March 2020 at Rajaei Heart Center, Tehran, Iran. The study received approval from the Ethics Committee of Tehran Heart Center with the ethics code IR.TUMS.THC.REC.1401.027.

### 2.2. Inclusion Criteria

Patients with previously confirmed *β*-thalassemia major on chelation therapy who had undergone at least two CMR evaluations were retrospectively included. Although T2^*∗*^ MRI is an age-dependent procedure, we did not impose any age restrictions on patient inclusion in the study. Our protocol explicitly stated that general anesthesia was considered for noncompliant patients or those under six years old. Based on previous suggestions [9], CMR evaluations were performed annually. They were performed with shorter intervals in the patients at high risk for cardiac complications and with longer intervals in the patients with stable conditions. Patients with more than two CMR evaluations during the study period had only the first and the last CMRs considered. Patients with heart failure were included only in case of taking the medications as prescribed by the guidelines.

### 2.3. Exclusion Criteria

We did not include patients with acute infections at the time of CMR evaluations and those without a baseline documented electrochemiluminescence assay of serum ferritin level (ECL, Roche Diagnostics, Basel, Switzerland). Patients with congenital and structural heart diseases, cardiomyopathy, myocarditis, or any cardiac dysfunction or altered anatomy unrelated to thalassemia were not included. Patients with systemic conditions other than thalassemia that could affect the myocardium and cardiac function were also excluded. These conditions included rheumatologic, infiltrative, metabolic, and storage diseases.

### 2.4. Measurement of the MRI and CMR Variables

The MRI sequences were gradient echo with T1-weighted (T1W) and T2-weighted (T2W) imaging. CMR was conducted using a 1.5 Tesla Siemens Avanto scanner (Siemens Medical Solutions, Erlangen, Germany), employing a single breath-hold multiecho technique for T2^*∗*^ assessment as described elsewhere [15]. General anesthesia was considered for noncompliant patients or those under six years old. Three short-axis views of the left ventricle were acquired to assess myocardial iron overload. Global myocardial T2^*∗*^ values were separately measured by two expert radiologists and a cardiologist and the data were rechecked in case of disagreement. The T2^*∗*^ sequence's cutoff values for myocardial siderosis were set at 20 ms (normal), 10–20 ms (moderate siderosis), and below 10 ms (severe siderosis). Liver T2^*∗*^ sequences for iron deposition were measured on a scale by which T2^*∗*^ lower than 2.1 was defined as severe iron overload and T2^*∗*^ over seven as mild liver toxicity. We used this formula (=0.202 + 25.4/liver T2^*∗*^) to calculate the liver iron concentration (mg/gram of dry weight), as previously described in the literature [16].

As indicated by the previous studies [17, 18], we calculated cardiac R2^*∗*^ with another formula (=1000/T2^*∗*^) to evaluate the lag between myocardial and liver iron unloading in the patients on iron chelation therapy. Since only two CMR evaluations were considered for each patient in the present study, we substituted the area under the curve (AUC) in the analyses with the proportional changes of myocardial R2^*∗*^ and liver iron concentration to estimate the mentioned lag. A positive time lag value indicated delayed myocardial iron unloading, negative values supported delayed liver iron unloading, and a time lag of zero indicated no delay.

Coronal, axial, and sagittal steady-state free precession sequences were employed to assess the functioning of both ventricles and the presence of thoracic and upper abdominal extramedullary hematopoietic tissue. Normal systolic function was defined as an ejection fraction of ≥60%, and the ejection fraction values below the mentioned cutoff were described as ventricular failure. Outcomes were based on the comparative changes in myocardial and liver iron T2^*∗*^ values, systolic myocardial dysfunction assessed by CMR, and mortality during the follow-up.

### 2.5. Statistical Analysis

Descriptive statistics were used for quantitative variables. The Wilcoxon signed-rank test was used to compare the two evaluations, as most variables were not normally distributed according to the results from the Shapiro–Wilk test. The correlation between the variables in each follow-up was evaluated by Spearman's rho test. The correlation coefficients with *r* smaller than 0.39 were regarded as mild, between 0.4 and 0.59 as moderate, and larger than 0.6 as strong relationships. The significance level was determined at a *p* value of ≤0.05. The analysis was performed using SPSS version 16.0.

## 3. Results

### 3.1. Baseline Characteristics

Out of 1023 *β*-thalassemia major patients who were referred for CMR assessment of iron overload, 55 individuals meeting the inclusion criteria were enrolled, with a mean age of 24.62 ± 7.94 years. Five patients (9%) had more than two CMR evaluations in the study period. During that period, alternative chelation therapies, as opposed to deferoxamine, were neither readily accessible nor financially feasible for most of the patients in our country, primarily due to their exorbitant cost. Consequently, all the included patients exclusively received deferoxamine as their iron-chelating medication. It is noteworthy that out of 1023 patients, only 34 were administered other chelators. Among these 34 patients, only one underwent at least two CMR sessions during the follow-up period and exhibited no congenital or structural heart diseases, cardiomyopathy, myocarditis, or any cardiac dysfunction or altered anatomy unrelated to thalassemia. Consequently, we excluded that patient from our final analyses to have a homogenous sample that can assess the impact of one chelator on cardiac and liver siderosis. Moreover, all patients demonstrated good compliance during the MRI studies, and none required general anesthesia. In addition, it is important to mention that there were no patients under six years old in our study. The baseline characteristics of the patients are outlined in Table 1. The mean follow-up duration was 24.3 ± 12.9 months, and mortality occurred in three patients (5.5%) during this period. The mean ferritin level was 1475.75 ± 771.12 ng/mL (1627.62 ± 780.45 and 1372.87 ± 760.08 in women and men, respectively).

### 3.2. Iron Clearance Lag

**Figure 1:**
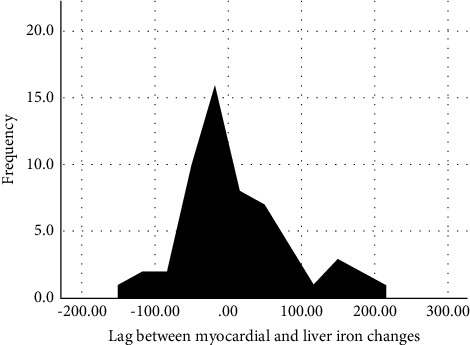
Lag between myocardial and liver iron changes. Positive values indicate delayed myocardial iron unloading, while negative values suggest delayed liver iron unloading. Based on the charts and their parameters (skewness = 0.870 (standard error = 0.322); mean = 5.91 (standard error = 9.24); median = −10.46; standard deviation = 68.49), the hypothesis of a significant difference between the distribution of the positive and negative values of the time lag was rejected (*p* value = 0.419).

Among the participants, 31 (56%) exhibited a positive lag (myocardial iron clearance slower than liver), while 24 (44%) had a negative lag (liver iron clearance slower than myocardium).  Figure 1 illustrates the distribution of the calculated lag. The lagged iron clearance of the myocardium was not significantly higher than that of the liver (*p* value = 0.419; skewness = 0.870 (standard error = 0.322); mean = 5.91 (standard error = 9.24); median = −10.46; standard deviation = 68.49). The lag between myocardial and liver iron clearance negatively correlated with baseline myocardial R2^*∗*^ (*p* value <0.001), the duration of the follow-up (*p* value <0.01), and the injections of deferoxamine (*p* value <0.05). In addition, a positive correlation was observed between the lag and the presence of extramedullary hematopoiesis (*p* value <0.05).

### 3.3. Cardiac Function and Iron Changes

As shown in Table 2, the ejection fraction of both ventricles significantly decreased during the follow-up. Right atrial diameter and right ventricular end-diastolic volume index (RVEDVI) significantly increased, while left atrial diameter and left ventricular end-diastolic volume index (LVEDVI) showed no significant changes. In terms of myocardial and liver T2^*∗*^ values, liver T2^*∗*^ significantly increased, while myocardial T2^*∗*^ displayed a nonsignificant rise during the follow-up. Liver iron concentration and myocardial R2^*∗*^ decreased during the follow-up (*p* value <0.05 and < 0.01, respectively).

### 3.4. Correlation Analysis

**Figure 2:**
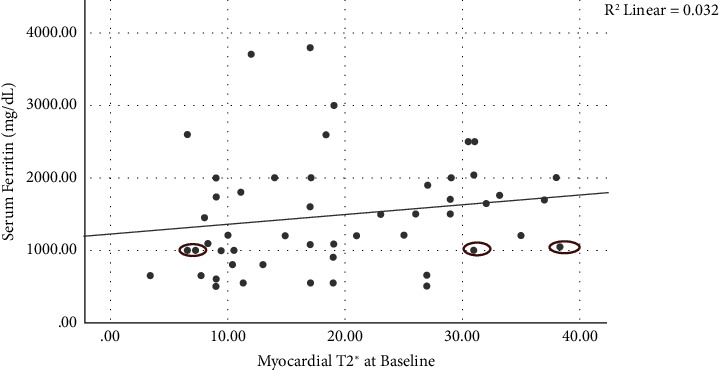
Simple scatter with a fit line of serum ferritin (mg/dL) by myocardial T2^*∗*^ at baseline.

As illustrated in Table 3, baseline myocardial T2^*∗*^ negatively correlated with the weekly number of deferoxamine injections (*p* value <0.01) and the timing between transfusions (*p* value <0.05). Positive correlations were observed between baseline myocardial T2^*∗*^ and serum ferritin (*p* value <0.05, Figure 2) and the presence of extramedullary hematopoiesis (*p* value <0.01). Patients with extramedullary hematopoiesis had significantly higher myocardial T2^*∗*^ than those without (mean ± SD: 23.34 ± 9.63 vs. 14.77 ± 8.79; *p*-value = 0.001). The correlation between baseline myocardial T2^*∗*^ and the left ventricle ejection fraction (LVEF) was moderate. Another moderate correlation was found between the baseline myocardial R2^*∗*^ and the time lag between myocardial and liver iron clearance. Except for the aforementioned correlations, the others detected were poor.

## 4. Discussion

Cardiac siderosis is one of the complications of *β*-thalassemia major that, with a respective estimated prevalence of 10% and 9%, may lead to heart failure and arrhythmia. Arrhythmia may present with atrial or ventricular origin and can induce sudden death following long QT intervals and torsade-de-pointes [9]. Thus, appropriate use of iron-chelating therapy in *β*-thalassemia major can mitigate myocardial dysfunction, reducing mortality and morbidity [14]. In this study, deferoxamine was exclusively employed as the chelating medication due to the limited accessibility and financial infeasibility of oral chelation therapy for most of the patients in our country at that time, mainly attributable to its exorbitant cost. Deferiprone and deferasirox are two other effective iron chelators that, contrary to deferoxamine, do not require parenteral administration. Previous findings suggest the low potential of deferoxamine in ameliorating cardiac iron overload [19, 20]. In addition, it is suggested that deferoxamine alone does not improve cardiac function, contrary to deferoxamine combined with deferiprone [9, 21, 22]. In line with the previous findings, the changes in myocardial T2^*∗*^ were insignificant in the follow-up of this study, although myocardial R2^*∗*^ was reduced in the follow-up.

Hepatic siderosis is another critical complication in *β*-thalassemia that is associated with hepatic injury, fibrosis, cirrhosis [23], and hepatocellular carcinoma. Thus, follow-up assessments of hepatic iron overload are strongly recommended [23, 24], with MRI assessments being the best surrogate of the iron content [25]. In response to the chelation therapy, a meaningful reduction was seen in liver iron concentration, although it was not associated with the changes in myocardial T2^*∗*^. The effect of deferoxamine on liver siderosis [19, 20] and its association with cardiac siderosis [26, 27] are controversial. Chen et al. indicate a correlation between myocardial T2^*∗*^ and liver iron concentration in the context of prevalent severe liver iron overload [26]. Half of our patients had severe liver iron overload. Yet, such a correlation was not observed. Regarding the effect of deferoxamine on liver siderosis, Ansari et al. revealed no noticeable improvement in myocardial and liver T2^*∗*^ after 12 months of therapy with deferoxamine [20]. Contrastingly, a review suggested the higher potentiality of deferoxamine compared to deferasirox and deferiprone in reducing liver iron overload [19].

Considering the mentioned findings, the myocardial-liver iron loading and clearance do not seem to follow a linear trend. Thus, predicting myocardial iron overload (and the subsequent cardiovascular complications) based on the surrogates of liver iron concentration could be misleading. Myocardial iron siderosis is expected to be mild when mild liver siderosis is present. However, variable values of myocardial siderosis are observed when moderate or severe liver siderosis is present [17, 26]. The mentioned variabilities can lead to a myocardial-liver lag in iron loading and clearance, as suggested by some previous studies on patients taking deferoxamine [17, 18, 26, 28].

The lag in the myocardial-liver iron loading could be attributed to the iron entrapment capability of the liver, reducing the myocardial accessibility to nontransferrin-bound iron. However, once liver injury and iron overconcentration occur at higher levels of liver iron concentration, myocardial iron overload is expected at variable stages [26]. Another lag mechanism is due to different clearance mechanisms following chelation therapy [17, 28]. Contrary to the liver, the slow myocardial degradation and diffusion of ferritin and hemosiderin and the lack of a facilitated deferoxamine uptake pathway result in a slower myocardial iron clearance than that of the liver [28]. The current study did not demonstrate such findings in the patients. According to Figure 1, the study patients were almost equally distributed among the groups demonstrating a positive and negative lag. Patients with extramedullary hematopoiesis and elevated baseline myocardial R2^*∗*^ were more likely to come up with delayed iron unloading of the myocardium than that of the liver, although this delay diminished with prolonged follow-up durations. Future studies are required to further confirm these findings and clarify the mechanisms leading to the lag mentioned before.

Myocardial T2^*∗*^ exhibited positive correlations with several assessed parameters, including extramedullary hematopoiesis, serum ferritin, and the ejection fraction of both ventricles. A similar correlation was found between the changes in myocardial T2^*∗*^ and LVEF. The mentioned correlations of extramedullary hematopoiesis [29, 30] and the ejection fraction of both ventricles [7, 27, 31] were in line with the previous findings. The association of higher cardiac T2^*∗*^ (i.e., lower cardiac iron content) with the presence of extramedullary hematopoiesis could be justified with the recruitment of iron in the sites of extramedullary hematopoiesis and the subsequently limited cardiac access to iron [30]. Contrary to the mentioned associations, the positive correlation of serum ferritin with myocardial T2^*∗*^ was not in line with previous studies [27, 31]. While serum ferritin is easily accessible, its reliability as a predictor of cardiac siderosis remains controversial [16, 31]. Several reasons advocate against relying solely on serum ferritin for predictions, emphasizing the need to explore alternative methods, such as cardiac magnetic resonance. One primary rationale stems from the scattered distribution of cardiac T2^*∗*^ based on serum ferritin, where distinct cardiac T2^*∗*^ values coexist despite similar serum ferritin levels (the circled values in Figure 2). In addition, ongoing controversies regarding this correlation further underscore the limitations and potential inaccuracies associated with using serum ferritin as an exclusive predictor of cardiac siderosis.

While interpreting the cardiac ejection fraction and volumetric indices in *β*-thalassemia major, the pathophysiology of cardiac dysfunction in thalassemia must be taken into account. The initial response to anemia of *β*-thalassemia is an increased volume overload and preserved or increased left ventricular ejection fraction (LVEF). Consequently, and in agreement with the previous studies [7, 31], we proposed a threshold of 60% to define normal LVEF in the patients. However, LVEF declines as *β*-thalassemia major progresses over time. The ejection fraction of both ventricles and the volumetric indices of the right ventricle statistically deteriorated. Nevertheless, the deteriorations were clinically minimal and insignificant for the abovementioned cardiac indices (Table 2), suggesting the hypothesis that deferoxamine may not necessarily lead to the clinical exacerbation of the patient's cardiac function. The prevalence of left-sided heart failure was 51.4% at baseline and 61.8% during the follow-up.

When assessing heart failure in *β*-thalassemia major, it is crucial to recognize that the pathophysiology of systolic myocardial dysfunction in these patients is multifactorial. Iron infiltration of the myocardium is the primary etiology of heart failure and arrhythmia in *β*-thalassemia major, with cardiac T2^*∗*^ significantly correlating with the two mentioned complications [6, 9]. Myocardial iron infiltration can alter the function of sodium and calcium channels, disrupting electrical conduction while sparing the cardiac conduction system [9]. Arrhythmia in thalassemia can result from both the deposition and chelator-mediated removal of cardiac iron [7]. Nevertheless, other unrelated etiologies may also result in heart failure and arrhythmia. Many of these concomitant issues are not revealed by chelation therapy and are persistent during the treatment cycle.

Most *β*-thalassemia major patients experience severe anemia, resulting in tissue hypoxia and compensatory volume overload. This volume overload increases the cardiac afterload, adversely affecting systolic function and ultimately culminating in heart failure [7, 8]. Volume overload can also result in arrhythmia [9]. Hypoxia can instigate a proatherogenic state, leading to cardiac ischemia and, eventually, heart failure. Microvasculopathy resulting from endothelial dysfunction and arteriosclerosis is one of the primary etiologies of heart failure in *β*-thalassemia major. Some mechanisms leading to microvasculopathy include hemolysis-induced nitric oxide deficiency, reduced vascular elasticity, and the hypercoagulable state caused by irregular erythrocytes and immunoinflammatory components [7, 8]. Among the other etiologies of heart failure in *β*-thalassemia major are pulmonary hypertension [23], valvulopathy, and postviral myocarditis due to thalassemia-associated immunocompromisation [7, 8]. As a result, heart failure in some *β*-thalassemia major patients may be independent of ventricular ejection fraction. Further studies are encouraged to discern the genuine role of each etiology in heart failure among patients with *β*-thalassemia major.

### 4.1. Strengths and Limitations

The present study longitudinally evaluated myocardial and liver iron loading and their predicting factors in an Iranian population suffering from *β*-thalassemia major. However, the study has several limitations, including a small sample size, a single-center setting, a relatively short follow-up duration, and a lack of an evaluation of other potential etiologies of heart failure in the studied patients. Since only one value was recorded for each CMR parameter, we could not calculate the interobservatory variability and intraobservatory variability for CMR parameters. Based on the mentioned limitations and the relatively small correlation coefficients, caution has to be taken to generalize the results.

## 5. Conclusion

Chelation therapy with deferoxamine in *β*-thalassemia patients effectively reduced the liver iron concentration and decreased myocardial R2^*∗*^. However, it did not significantly affect myocardial T2^*∗*^. Consequently, recruiting alternative therapies or their combination with deferoxamine may be necessary for improved outcomes. In addition, the study underscores the significant reliability of MRI findings compared to laboratory parameters, such as ferritin levels, in diagnosing and managing cardiac or liver iron overload. Therefore, there is a pressing need to establish health equity globally, with a specific focus on low-to-middle-income countries, ensuring that patients have access to the latest accurate diagnostic and treatment measures.

## Figures and Tables

**Table 1:** Basic characteristics of the study patients.

	(*N* = 55)
Male, number (%)	32 (58.2)
Age (years), mean (SD)	24.62 (7.94)
Splenectomy, number (%)	20 (36.4)
Mortality, number (%)	3 (5.5)
Duration of follow-up (months), mean (SD)	24.38 (12.97)
Extramedullary hematopoiesis at baseline, number (%)	23 (41.8)
Extramedullary hematopoiesis during follow-up, number (%)	24 (43.6)
Ferritin (ng/mL), mean (SD)	1475.75 (771.12)
Right ventricular failure at baseline, number (%)	33 (60.0)
Right ventricular failure during follow-up, number (%)	43 (78.2)
Left ventricular failure at baseline, number (%)	31 (56.4)
Left ventricular failure during follow-up, number (%)	34 (61.8)
Myocardial siderosis at baseline, number (%)	35 (63.6)
Moderate myocardial siderosis, number (%)	20 (36.4)
Severe myocardial siderosis, number (%)	15 (27.3)
Myocardial siderosis during follow-up, number (%)	32 (58.2)
Moderate myocardial siderosis, number (%)	26 (47.3)
Severe myocardial siderosis, number (%)	4 (7.3)
Liver iron concentration at baseline (mg/gram of dry weight), mean (SD)	16.39 (12.80)
Liver iron overload at baseline, number (%)	48 (87.3)
Moderate liver iron overload, number (%)	20 (36.4)
Severe liver iron overload, number (%)	28 (50.9)
Liver iron concentration during follow-up (mg/gram of dry weight), mean (SD)	13.87 (9.61)
Liver iron overload during follow-up, number (%)	49 (89.1)
Moderate liver iron overload, number (%)	22 (40)
Severe liver iron overload, number (%)	27 (49.1)

**Table 2:** Changes in cardiac parameters and iron deposition indices during follow-up.

Parameter	Mean (SD) at baseline	Mean (SD) during follow-up	*p* value (exact)
Right atrial diameter	17.24 (6.00)	18.44 (6.54)	0.050^a^
Left atrial diameter	20.09 (4.70)	19.94 (5.84)	0.970
Left ventricular ejection fraction	56.46 (10.36)	54.31 (11.49)	0.032^a^
Right ventricular ejection fraction	54.90 (10.64)	50.58 (10.99)	<0.001^b^
Right ventricular end-diastolic volume index	76.73 (22.08)	79.43 (24.17)	0.026^a^
Left ventricular end-diastolic volume index	80.13 (19.88)	81.04 (21.19)	0.778
Liver T2^*∗*^	18.35 (10.02)	20.35 (8.09)	0.011^a^
Liver iron concentration	16.39 (12.80)	13.87 (9.61)	0.010^b^
Myocardial T2^*∗*^	2.98 (2.44)	3.28 (2.53)	0.198
Myocardial R2^*∗*^	77.63 (52.59)	60.30 (35.68)	0.006^b^

^a^Significant at *p* ≤ 0.05. ^b^Significant at *p* ≤ 0.01.

**Table 3:** Correlations between baseline and variations of myocardial T2^*∗*^ and liver iron concentration and that between baseline and variations in other variables.

Correlations with baseline measures and differences		Correlation coefficient	*p* value
Baseline myocardial T2^*∗*^ (correlation with the baseline measures)	Serum ferritin	0.280	0.044^a^
	Transfusion timing (baseline)	−0.288^a^	0.039^a^
	Weekly deferoxamine	−0.375^b^	0.006^b^
	Extramedullary hematopoiesis	0.424^b^	0.001^b^
	Splenectomy	0.033	0.811
	Baseline liver iron concentration	−0.063	0.645
	Baseline left ventricular ejection fraction	0.512^b^	<0.001^b^
	Baseline right ventricular ejection fraction	0.445^b^	0.001^b^

Changes in myocardial T2^*∗*^ (correlation with the difference of the two measures)	Liver iron concentration	0.153	0.264
	Right atrial diameter	−0.036	0.834
	Left atrial diameter	0.218	0.195
	Left ventricular ejection fraction	0.278^a^	0.044^a^
	Right ventricular ejection fraction	−0.076	0.584
	Right ventricular end-diastolic volume index	−0.427^b^	0.002^b^
	Left ventricular end-diastolic volume index	−0.257	0.061

Baseline liver iron concentration (correlation with the baseline measures)	Serum ferritin	0.192	0.173
	Transfusion timing (baseline)	−0.095	0.503
	Weekly deferoxamine	0.043	0.764
	Extramedullary hematopoiesis	−0.184	0.180
	Splenectomy	0.030	0.832

Lag between myocardial and liver iron changes	Baseline myocardial R2^*∗*^	0.608^b^	<0.001^b^
	Baseline liver iron concentration	0.127	0.355
	Serum ferritin	0.076	0.594
	Transfusion timing (baseline)	−0.112	0.430
	Duration of the follow-up	−0.377^b^	0.005^b^
	Weekly deferoxamine	−0.295^a^	0.034^a^
	Extramedullary hematopoiesis	0.276^a^	0.041^a^
	Splenectomy	0.155	0.263

^a^Significant at *p* < 0.05. ^b^Significant at *p* < 0.01.

## Data Availability

The dataset used and analysed during the current study are available from the corresponding author on reasonable request.

## Supplementary Materials

Supplementary Materials The filled Strengthening the Reporting of Observational Studies in Epidemiology Statement (STROBE Statement) Checklist for this paper can be found in the Supplementary Materials.
